# Celiac Disease: Beyond Diet and Food Awareness

**DOI:** 10.3390/foods14030377

**Published:** 2025-01-24

**Authors:** Lourdes Herrera-Quintana, Beatriz Navajas-Porras, Héctor Vázquez-Lorente, Daniel Hinojosa-Nogueira, Francisco J. Corrales-Borrego, Maria Lopez-Garzon, Julio Plaza-Diaz

**Affiliations:** 1 Department of Physiology, Schools of Pharmacy and Medicine, University of Granada, 18071 Granada, Spain; hectorvazquez@ugr.es; 2 Department of Endocrinology and Nutrition, Foundation for the Promotion of Health and Biomedical Research in the Valencian Region (FISABIO), University Hospital Doctor Peset, 46017 Valencia, Spain; beatriz.navajas@fisabio.es; 3 Unidad de Gestión Clínica de Endocrinología y Nutrición, Laboratorio del Instituto de Investigación Biomédica de Málaga (IBIMA), Hospital Universitario de Málaga (Virgen de la Victoria), 29010 Málaga, Spain; daniel.hinojosa@ibima.eu; 4 School of Health Sciences, Universidad Internacional de La Rioja, Avenida de la Paz, 137, 26006 Logroño, Spain; fisiofjcorrales@gmail.com; 5 Biomedical Group (BIO277), Department of Physical Therapy, Health Sciences Faculty, University of Granada, 18171 Granada, Spain; maloga@ugr.es; 6 Instituto de Investigación Biosanitaria IBS.GRANADA, Complejo Hospitalario Universitario de Granada, 18014 Granada, Spain

**Keywords:** celiac disease, gluten-free diet, autoimmune disorder, dietary management, autoimmune response, genetic predisposition, health advocacy

## Abstract

Celiac disease is attributable to a combination of genetic predisposition and exposure to dietary gluten, with immune system involvement. The incidence is increasing globally, and the societal economic burden of celiac disease stretches beyond the cost of gluten-free food. This enteropathy that affects the small intestine has been related to different disorders and comorbidities. Thus, the implications of suffering from this disease are multidimensional and need further consideration. Celiac disease is a serious condition that remains under-recognized, resulting in an increased need for programs for better management. This review aims to summarize the current evidence regarding celiac diseases, with special emphasis on clinical implications, diagnosis, dietary management, socioeconomical aspects, and future perspectives.

## 1. Introduction

### 1.1. Celiac Disease Overview

Those genetically predisposed to celiac disease (CD) suffer from a chronic immune-mediated enteropathy affecting the small intestine as a result of gluten consumption [1]. This condition is characterized by a permanent immune-mediated response to gluten, which is found in wheat, barley, rye, and their derivatives [2]. Furthermore, a considerable number of processed foods may contain gluten as an additive or thickener, even if the primary ingredients are not derived from these grains. In addition to this, cross-contamination with gluten represents an additional potential hazard, particularly in circumstances where different foodstuffs are handled simultaneously during the food processing stage [3].

In general terms, gluten is defined as the largely proteinaceous mass that remains after washing a dough made from wheat flour. Nevertheless, related proteins present in other cereals are known as gluten as well; the term used, “prolamins”, is more correct when referring to all these proteins [4]. Gluten is considered one of the most complex protein aggregates in nature, being formed by gliadins (important for dough viscosity) and glutenins (responsible for dough elasticity), which are the storage proteins of the grain. Namely, gliadins from wheat gluten comprise ω5-gliadins, ω1,2-gliadins, α-gliadins, and γ-gliadins, and glutenins consisting of low and high molecular weight glutenin subunits [5].

The clinical manifestations of gluten exposure in individuals with CD exhibit a broad spectrum of characteristics, which collectively serve to define this condition as a multisystem disorder rather than an isolated intestinal disease [6]. It is characterized by damage to the small intestine and the presence of specific antibodies, resulting in the malabsorption of nutrients and a variety of gastrointestinal symptoms, including chronic diarrhea, bloating, weight loss, fatigue, and iron deficiency anemia. Furthermore, it presents with extraintestinal symptoms, including dermatitis, osteoporosis, neuropathies, and reproductive disorders [7].

The primary treatment is a lifelong strict gluten-free diet (GFD), which has been demonstrated to result in the recovery of intestinal mucosa and the resolution of symptoms in the majority of patients. Nevertheless, it is imperative to conduct periodic medical follow-ups to ascertain the patient’s compliance with the dietary regimen and to identify potential complications, such as refractory CD or intestinal lymphoma [8].

The interest about CD has arisen over the years. The review of the Web of Science scientific database, conducted up to September 2024, revealed the existence of approximately 17,806 articles pertaining to the subject of CD. Over the past two decades, the number of articles published annually has increased from 300 in 2003 to over 700 in 2023. A geographical analysis of the data reveals that the USA is the leading country in terms of research output, accounting for 22.4% of the total. Subsequently, Italy and England account for 15.4% and 8.7% of the total, respectively. These findings are in accord with the trends identified by Dermir and Comba [9].

### 1.2. Prevalence and Epidemiology

The first documented case of CD is believed to have been described by the ancient Greek physician Aretaeus. However, it has not been until recent years when the incidence of gluten-related disorders has been reported to increase markedly, rendering them a significant public health concern [10].

In recent decades, CD has gone from being considered a rare disease to being one of the most common life-long disorders, with an estimated prevalence in the general population of 0.7–2.9%—this frequency being higher in females, patients with autoimmune disorders, and in relatives of CD-affected individuals [11]. Furthermore, projections indicate that not only is the prevalence of the disease on the rise, but so too is its incidence, with an estimated annual increase of 7.5% over past years [12]. However, there is still a need for studies in this respect in many countries, as it is of interest that the results of prevalence vary depending on the diagnostic criteria. As a result, CD prevalence has been estimated at 1.4% based on serologic test results, as well as 0.7% based on biopsy results [13].

The prevalence of the disease is higher in Europe, Oceania, and North America than in other regions (with disparities among data from distinct countries), which may be attributed to differences in diet and under-diagnosis. However, recent studies have demonstrated an increasing incidence in Asia, Africa, and Latin America. This may be attributed to alterations in dietary patterns and heightened awareness of the disease [12,13,14]. A case in point is the higher rates reported by countries such as Finland and Sweden in comparison to the rest [13].

Furthermore, there are significant ethnic and racial variations in the prevalence of celiac disease. For example, data from the National Health and Nutrition Examination Survey in the USA indicate that the prevalence of CD is lower in non-Hispanic Black and Hispanic populations than in white individuals [15]. Prevalence can also vary even within the same country. For instance, in India, despite a similar distribution of haplotypes between the northern and southern regions, the Punjab region exhibits a higher prevalence of the disease than the south [2].

On the other hand, certain pathologies may be related to an increased prevalence of this disease. For example, individuals with type 1 diabetes are at higher risk of suffering from another autoimmune disease, such as CD [16]. A comparable phenomenon has been observed in other diseases, such as non-alcoholic fatty liver disease or metabolic syndrome, which are frequently attributed to the adoption of a GFD [17]. In fact, causal relationships between CD and autoimmune diseases have been reported. Namely, the analysis of single nucleotide polymorphisms in the European population found significant associations with 13 autoimmune diseases (e.g., Graves’ disease, asthma, psoriasis, Crohn’s disease, type 1 diabetes, rheumatoid arthritis, etc.) [18].

Recent studies indicate that, in general, the increase in prevalence is due to three main factors: increased awareness of the disease, improved diagnostic and screening methods, and increased knowledge of a wide range of factors that influence disease expression. Such factors include alterations in dietary patterns, early gastrointestinal infections, and modifications in the gut microbiota [2]. Nevertheless, the prevalence remains uncertain due to the significant clinical variability associated with the disease. In a considerable number of cases, the disease remains undiagnosed in most countries, resulting in a high prevalence of undiagnosed or asymptomatic CD [11,19].

### 1.3. Celiac Disease in Specific Populations

CD is a condition that affects different population groups in diverse ways, exhibiting a wide range of clinical presentations that vary according to age, gender, and other physiological factors. For example, epidemiological studies have shown that women are diagnosed almost twice as frequently as men [12]. The observed increase may be attributed to hormonal or genetic variations or an elevated predisposition to pursue medical attention [20]. Furthermore, women with CD may also have an increased risk of experiencing reproductive complications, including infertility and recurrent miscarriages [21]. In contrast, male patients are frequently misdiagnosed due to their lower propensity to undergo a duodenal biopsy examination [2].

Although historically regarded as a pediatric disorder, CD can manifest at any age, with an increasing number of diagnoses being made in adults and the elderly [22,23]. These diagnoses do not necessarily indicate a late discovery; rather, they could be the result of a loss of gluten tolerance, which typically occurs in adulthood. Nevertheless, recent prospective cohort studies have demonstrated that the majority of patients develop CD before reaching the age of 10 years [2]. In children, CD typically presents with characteristic symptoms, including chronic diarrhea, bloating, and stunted growth. In adults, however, the disease is more challenging to diagnose due to the greater diversity of its clinical manifestations [24].

In pediatric populations, previous research has investigated the potential role of early gluten introduction and duration of breastfeeding as possible risk factors for the development of CD [25]. Generally, the disease is particularly critical in children due to its impact on growth and development. Consequently, there is a strong focus on early detection and management to prevent long-term complications [26]. The presentation of CD in older populations may be characterized by subtle or atypical symptoms, which can complicate the diagnostic process. The presence of comorbidities can further contribute to an under-diagnosis of CD in this population, leading to an increased risk of associated morbidity [27].

The prevalence of CD is higher in other vulnerable populations with disorders such as Down’s syndrome or Turner’s syndrome. Therefore, regular follow-up is necessary [16]. Furthermore, other populations with specific physiological conditions, such as pregnant women with undiagnosed or poorly controlled celiac disease, have been demonstrated to be at elevated risk of developing obstetric complications, including miscarriages and low birth weight. This highlights the necessity for the development of targeted screening strategies [28].

## 2. Understanding the Pathogenesis of Celiac Disease

CD is a serious and under-recognized condition, partly because the boundaries of this disease are not always clear, and a consensual diagnostic criterion of CD and related conditions does not exist [1]. One of the highly reliable parameters for the diagnosis of CD is specific serology, with discrepancies between experts regarding the need of intestinal biopsies to confirm that diagnosis. Overall, there is a trend to avoid biopsies in children when levels of anti-tissue transglutaminase IgA antibody (anti-tTG-IgA) exceed >10 times the upper limit. However, this approach has not been formally implemented in all countries [29].

The ingestion of cereals containing gluten is generally associated with CD, but there are other pathological conditions caused by this cereal consumption, such as gluten sensitivity or wheat allergy. In summary, the main gluten reactions can be classified in the following categories: autoimmune (CD, gluten ataxia, and dermatitis herpetiformis), allergic (wheat allergy), and other possibly immune-mediated reactions (gluten sensitivity) [30]. Moreover, evidence and knowledge has grown in recent years, showing more disturbances related to gluten than classically recognized, using the term “gluten-related disorders” to encompass the wide variety of conditions [31].

Gluten sensitivity is referred to as “reactions to gluten with no implication of neither autoimmune nor allergic mechanisms”, while both CD and wheat allergy are mediated by T cells in the gastrointestinal tract [30]. Regarding wheat allergy and intolerances, the allergenic reactions are classified as (I) immunoglobulin (Ig)E-mediated responses, where IgE binds to allergens resulting in the release of immune and inflammatory mediators, and (II) non-IgE-mediated responses, which are associated with the formation of immune complexes between antibodies and food components. In these disorders, different protein components may be involved (e.g., prolamins, gliadins, glucoproteins, profilins, etc.) [32]. For its part, CD originally was thought to be a hypersensitivity disorder; however, currently it is known to be a tissue-specific autoimmune disease. CD shows similarities with other autoimmune diseases such as type 1 diabetes, being characterized by typical autoimmune features (i.e., human leukocyte antigen (HLA) association, specific autoantibodies, and immune-mediated destruction of enterocytes [33].

CD is a complex inflammatory disorder with a multifactorial etiology. It is known that there exists an important genetic background influencing its development. Different genes appear to contribute to CD occurrence, the most important being the HLA gene. Most individuals with CD show specific HLA-DQ allotypes, related to, namely, HLA-DQ2 and HLA-DQ8 haplotypes [34]. Although these factors are not sufficient to cause CD, DQ2 and DQ8 are of importance since they act as restriction elements for gluten-specific CD4+ T lymphocytes [35]. Another important factor in CD patients is the presence of specific IgA and IgG antibodies whose target is the autoantigen tissue transglutaminase 2 (TG2) [34,35]. Although there are intraepithelial lymphocytes residing in the gut epithelial cells in all individuals, the presence of gluten-specific CD4+ T cells is a common finding in patients with CD [34]. In summary, one of the main mechanisms implied in the pathogenesis of CD includes the HLA-DQ allotype variants which condition the antigen presentation to CD4+ T cells, leading to T cell reactivity against gluten peptides [33].

## 3. Clinical Manifestations and Consequences

### 3.1. Gastrointestinal Manifestations

The gastrointestinal manifestations of CD are numerous, being much more evident in children than in adults [36]. The most common manifestations include weight loss, chronic diarrhea, and abdominal pain [37]. The inflammation and duodenal dysfunction that occur during active CD have different adverse effects on the stomach [38]. In addition, stomach dysfunction occurs in the case of untreated CD, causing belching, indigestion, fullness, nausea, and even vomiting [39]. Gastritis and peptic ulcers are also common in patients with CD, both in children and adults. Similarly, chronic vomiting and nausea are usual during untreated CD, but on a GFD, they may disappear. However, in the case of gastritis and peptic ulcers, the pathogenesis is not entirely clear and more research should be conducted [39].

The intestine is the main organ responsible for the absorption of nutrients, water, and electrolytes. CD causes a reduction in the area of intestinal absorption [40]. The most common disorder of CD is chronic diarrhea, which takes the form of watery, loose stools that occur frequently. This is due to the inability to digest certain nutrients at the intestinal level, causing the colon to produce more water instead of absorbing it, leading to diarrhea [39,41].

In addition to intestinal and gastric symptoms, esophageal manifestations, such as gastro-esophageal reflux disease and eosinophilic esophagitis, have been observed [42]. Also at the pancreatic level, some research has indicated an association between exocrine pancreatic insufficiency and CD [43]. Additionally, around 10% of CD patients are diagnosed with a variety of hepatic and biliary disorders, including hepatitis B and C, autoimmune hepatitis, non-alcoholic fatty liver disease, liver cirrhosis or fibrosis, sclerosing cholangitis, and biliary cholangitis [44,45].

### 3.2. Nutrient Deficiencies

Currently, there is no specific tool to assess undernutrition in CD. Despite the fact that CD activity and adherence to the GFD are important factors influencing malnutrition [46], the available tools do not take these factors into consideration. A GFD excludes wheat, barley, and rye and carries many risks related to reduced acceptability and nutritional inadequacy [47]. Available gluten-free foods (GFFs) are generally low in fiber and some micronutrients compared to their gluten-containing counterparts [48].

Due to malabsorption, micronutrient deficiencies are common in patients with untreated CD. Therefore, nutritional assessment is very important in these cases and allows the identification of patients at risk of malnutrition [49]. Reduced micronutrient levels have been found in up to 88% of the adult population diagnosed with CD [50]. In addition, it has been shown that a GFD not only leads to micronutrient deficiencies but can also lead to excessive micronutrient intake [46].

Micronutrients absorbed in the proximal duodenum are at an increased risk of malabsorption as a result of CD. Nevertheless, other factors, such as a poor nutritional quality of the GFD or a lack of fortification in gluten-free products (GFPs) with calcium, folic acid, nicotinic acid, thiamine, and iron can lead to persistent micronutrient deficiencies regardless of disease activity [51]. The results of several studies indicate that adults with CD who follow a GFD have micronutrient intakes below the recommendations for vitamin D, iron, calcium, vitamin B12, thiamin, vitamin B6, folate, vitamin A, zinc, and magnesium. Additionally, children’s GFDs have been found to have low levels of iron, vitamin D, calcium, vitamin B12, vitamin B6, folate, zinc, and magnesium [50]. With a GFD, some of these nutritional deficiencies can be reversed, such as vitamin B12 and vitamin K. However, vitamin A, vitamin D, iron, copper, and zinc appear to only be partially reversed [50,52].

The adoption of a strict GFD for life is challenging; however, it is the only effective treatment for CD. As a result, all newly diagnosed patients with CD should receive a routine nutritional evaluation and GFD education from an experienced dietitian, as well as a follow-up for patients with persistently elevated or detectable celiac serologies, persistent symptoms, difficulty adhering to the GFD, or poor nutritional quality of the GFD [53,54].

### 3.3. Extraintestinal Symptoms and Comorbidities of Celiac Disease

If CD is not adequately treated, it may lead to some serious complications and comorbidities. CD presents with a wide range of clinical symptoms, including classical and non-classical gastrointestinal symptoms, extraintestinal manifestations, and subclinical manifestations [55]. Gastrointestinal manifestations are the most common and include flatulence, bloating, and chronic diarrhea [56]. Extraintestinal manifestations include decreased bone mineralization, osteopenia and osteoporosis, arthralgia, anemia, neurological symptoms, and fatigue [37]. Other comorbidities commonly associated with CD are hypertransaminemia, infertility, short stature, and delayed puberty [57].

Extraintestinal symptoms may sometimes be the only indicators of CD, leading to a significant delay in diagnosis. Some of the most frequently reported symptoms are fatigue, tooth enamel defects, and osteoporosis. In some patients, there may be only one or two symptoms, while in others, there may be a number of symptoms occurring at the same time [58]. Additionally, non-compliance with a strict GFD further contributes to the occurrence of these manifestations in patients with CD who have already been diagnosed. It is still unclear how the comorbidities of CD are caused by the precise pathophysiological mechanisms. There are some extragastrointestinal manifestations that are directly related to the malabsorption of essential nutrients, particularly minerals and vitamins [59].

Refractory CD is one of the complications. The condition is characterized by prolonged villous atrophy in duodenal biopsies from patients with CD [60]. Symptoms of malabsorption accompany this rare complication of CD, which has a variable prevalence and incidence. There has been a decrease in the incidence of refractory CD over the last 20 years, possibly as a result of increased awareness, stricter adherence to the GFD, and an increased availability of GFPs [61,62]. This condition may be primary, refractory at the time of diagnosis, or secondary, occurring following a period of response to GFD treatment [63].

In the oral cavity, both children and adults may suffer from late eruption of teeth, enamel defects, caries, plaque, and periodontitis [64]. There are numerous ocular manifestations associated with this disorder, including nyctalopia, dry eye, thyroid-related orbitopathy, cataracts, retinal vein occlusion, uveitis, and neuro-ophthalmic manifestations [65]. Dermatitis herpetiformis is commonly referred to as CD of the skin. Despite the fact that symptoms may not respond immediately to a GFD, patients with dermatitis herpetiformis must begin a lifelong regimen. In addition, CD may be related to a number of diseases, including Alzheimer’s disease, psoriasis, rosacea, androgenic alopecia, and atopic dermatitis [58,66]. A variety of neurological symptoms and alterations of the peripheral or central nervous system have been described in patients with untreated CD, including epilepsy, peripheral neuropathies, cerebellar ataxia, migraines, and cognitive impairment [34]. The pathogenesis and effects of a GFD should, however, be further investigated.

It is common for patients with CD to experience bone manifestations such as decreased bone pain, bone density and fractures, osteoporosis, and osteopenia. While several mechanisms have been proposed, such as malabsorption of calcium and vitamin D and the release of proinflammatory cytokines, the underlying mechanisms remain unclear [67]. CD has also been linked to joint and muscle conditions [68]. Additionally, CD has sometimes been associated with renal diseases, such as nephropathy or chronic kidney disease [69].

In addition, CD has also been related to intestinal malignancies, such as T-cell lymphoma, which in turn is related to small bowel and colorectal cancers and enteropathies [70]. Malignant neoplasms and an increased risk of mortality are also associated with CD [71]. One example is the development of small bowel cancer, where CD-associated small bowel carcinoma predominantly affects the jejunum after an average duration of CD of 17 months. Mismatch repair deficiencies are more common in small bowel carcinomas related to CD than in those associated with Crohn’s disease or sporadic small bowel carcinomas [72].

The most prevalent symptoms of undiagnosed CD in children and adults are abdominal pain, flatulence, chronic diarrhea, and bloating. In most cases, these problems can be resolved by following a strict GFD. Intestinal intussusception and intestinal malignancies are rare conditions that are rarely associated with CD. A greater amount of attention should be paid to them by physicians and further research should be conducted [39].

## 4. Diagnostic Approaches to Celiac Disease

### 4.1. Differential Diagnosis of Celiac Disease

Multiple factors play a role in the pathogenesis of CD, including environmental, immunological, and genetic factors. Consequently, diagnosis is based on a combination of criteria, including clinical, serological, genetic, and histological features. These may include the detection of anti-tissue transglutaminase antibodies or a small bowel biopsy showing villous atrophy and increased intraepithelial lymphocytes [6]. CD exhibits similar duodenal histopathological characteristics as numerous other intestinal disorders, such as irritable bowel syndrome, which leads to misdiagnosis in some cases [12]. Moreover, population-based screening studies have demonstrated that for each diagnosed case, there are numerous undiagnosed cases, thereby underscoring the necessity for more efficient detection strategies and more general screening [55]. Therefore, a thorough assessment of characteristic histological findings, clinical data, serological markers, and immunological factors is necessary for an accurate diagnosis [73].

Within the category of CD mimickers, various groups exist. One of them is infectious diseases. Giardiasis, which is brought on by *Giardia lamblia*, ranks as one of the most prevalent intestinal parasite infections [74]. It can induce intestinal villous atrophy [75]. Another example is gastritis caused by *Helicobacter pylori*, resulting in epithelial lymphocytosis and mild villous changes. The presence of foveolar gastric metaplasia and marked neutrophilic infiltration in the epithelium or lamina propria can distinguish *Helicobacter pylori*-associated peptic duodenitis from microscopic alterations in CD [76]. Finally, viral gastroenteritis can cause mucosal changes, but in these cases the mucosa recovers after infection [77].

Secondly, drugs such as non-steroidal anti-inflammatory drugs, immunomodulators, and antineoplastic drugs can histologically mimic CD. However, villous atrophy is rarely described in these cases [74]. In particular, olmesartan, an angiotensin II receptor blocker, causes partial to complete villous atrophy, thus mimicking the histology of CD [78].

Furthermore, during the diagnosis process, CD could be confused with other immunoinflammatory conditions. Food protein-related intestinal disorders, which have histological abnormalities resembling those of CD, are frequently transient or can be resolved by excluding the allergen from the diet. Pernicious anemia may lead to inadequate flattening of the intestinal villi and irritation of the mucosa [79]. Collagenous sprue, an uncommon disorder leading to inadequate absorption, is often misdiagnosed as CD. Yet, recognizing a dense layer of subepithelial type I collagen alongside inflammatory cells and captured capillaries can result in an accurate diagnosis [80]. Common variable immunodeficiencies may also mimic the features seen in CD. However, duodenal samples from individuals with CD may show two distinct features not commonly found in these patients: reduced plasma cells (observed in approximately two-thirds of cases) and increased follicular lymphoid hyperplasia [73]. Finally, autoimmune enteropathy is characterized by the thinning of the intestinal mucosa and the existence of autoantibodies attacking enterocytes and/or goblet cells. An increase in the lamina propria with a combination of inflammation and neutrophil infiltration, or a pattern resembling CD, is a distinguishing characteristic. It is important to note that biopsies from various parts of the digestive system often show histological abnormalities and can help with diagnosing issues.

### 4.2. Treatment/Medication

As of now, the only treatment for CD is the permanent and complete elimination of gluten from the diet. Gluten is the antigenic trigger for the celiac immune response. It is an effective treatment modality that may be able to reverse the specific alterations caused by CD, including the clinical symptoms that contribute to poor quality of life [81]. Despite overall mortality being higher in the celiac population, it appears that for those patients who can adhere to a GFD, there is no statistically significant difference compared to the healthy population [81,82].

Despite there being many reasons why adherence to the GFD must be strict to improve outcomes and it is supposed to be theoretically simple, this diet has many complexities and must not only be gluten-free, but also balanced, covering the total energy and nutritional requirements [83,84]. As for the disadvantages, it is important to note that following a GFD influences social issues, especially because it is an expensive diet and can be tedious to follow in various social settings [85]. There is little doubt that these reasons contribute to a substantial proportion of patients not adhering strictly to a GFD. Current results show that adherence to the GFD shows highly variable figures in the celiac population following a GFD [86]. Low adherence implies a risk for the people with CD themselves, as they are exposing their intestinal mucosa to gluten, inducing malabsorption effects and other risks [81]. In contrast, some patients who adhere to a strict diet still experience symptoms. Possible causes explaining this phenomenon include small intestinal bacterial overgrowth, transient lactose intolerance, pancreatic insufficiency, poorly absorbed short-chain carbohydrates, and gastrointestinal comorbidities [87].

Due to these reasons, non-dietary treatment options are of considerable interest as a means of preventing or treating this disease [88]. The pathophysiology of CD and the main therapeutic approaches are currently being pursued to develop CD therapies. The gluten is modified/neutralized by latiglutenase [89], zamaglutenase [90], and AGY 010 [91], and a strategy for diminishing gluten’s toxic effects has been suggested that involves reducing the immunogenic components of its peptides in the stomach [92]. There is currently an ongoing Phase IIb crossover study investigating the efficacy and safety of latiglutenase treatment in symptomatic seropositive patients with CD who are on a GFD while undergoing periodic gluten exposure (NCT04243551). An ongoing Phase II study with 377 patients with active CD attempting a GFD is testing zamaglutenase (NCT05353985). With the use of gluten-containing bars, the study is designed to mimic real-life inadvertent gluten exposures.

The oral enzyme therapy, on the other hand, is quite attractive, since immunogenic gluten peptides are the initiators of the pathogenic cascade. The first peptidase therapies used proteases, which proved to be highly effective in vitro. There has, however, been no consistency between the results of several in vivo studies [93,94,95]. This may be due to the acidic pH of the stomach and interference with other dietary components [96]. *Aspergillus* Niger’s prolyl endopeptidase (AN-PEP), an acid-resistant enzyme, is now commercially available (Tolerase G) [88].

The degradation of tissue transglutaminase 2 is another important approach [97]. The ratio of villus height to crypt depth has been shown to reduce mucosal injury in patients with CD. Currently, this is the first pharmacological approach that has shown signs of protecting against gluten-induced mucosal injury [98]. Deamidation by TG2 leads to increased HLA-DQ2/8 affinity, which leads to the activation and expansion of gluten peptide-specific CD4+ type 1 helper T cells and their release of inflammatory cytokines [99], and its inhibition may provide a novel therapeutic option. ZED1227 is an orally available, small molecule inhibitor of intestinal TG2. For six weeks, ZED1227 effectively attenuated intestinal mucosal injury in patients with CD who received a moderate daily dose of gluten (3 g/day) during a Phase II proof-of-concept study [100].

The strategy is to induce gluten tolerance [101]. As part of this approach, tolerogenic dendritic cells may be used that promote an anti-inflammatory environment, induce clonal anergy or the deletion of antigen-specific T cells, or promote the differentiation of antigen-specific regulatory T cells, inhibiting gluten-specific CD4 T cells in individuals with CD [102]. Nexvax2 sought to induce tolerance by dermal vaccination with three immunodominant gluten peptides dissolved in saline. During the study, patients on a GFD received multiple doses of NexVax2 or placebo, followed by a double-blind, placebo-controlled oral gluten challenge. Nevertheless, it did not prevent the development of villous atrophy and symptoms associated with CD after the gluten challenge [103,104,105].

A strategy that seeks to reestablish tolerance to gluten is extremely promising since it targets mechanisms responsible for CD pathology rather than downstream effects. Patients may be able to consume gluten through such strategies and benefit from a significant improvement in their quality of life. Another approach involves intravenous administration of nanoparticles encapsulating gluten protein extract (TAK-101) in order to ensure the presence of a wide variety of gluten epitopes. There is a possibility that this approach may be a promising treatment, but further research is necessary [106]. In a Phase IIa study (NCT05660109), TPM502 is currently being tested in adults with CD. The purpose of this placebo-controlled trial is to determine the safety and tolerability of two infusions of TPM502 at increasing doses in patients with well-controlled CD who have been on a GFD for at least six months.

Monoclonal antibodies can be used to target cytokines. A key cytokine in the pathogenesis of CD appears to be interleukin (IL)-15 [107]. Overexpression of IL-15 stimulates intraepithelial cytotoxic CD8 T cells, which results in the destruction of intestinal tissue. The first IL-15-targeted assay evaluated a monoclonal antibody (mAb) (AMG714) against IL-15 [108]. Clinical symptoms, particularly diarrhea, showed signs of improvement.

Larazotide acetate, a locally acting tight junction regulator used to decrease gut permeability, has reached Phase III development (CeDLara, NCT03569007). In addition to paracellular and transcellular pathways for gliadin transport, larazotide acetate might have compromised its therapeutic potential [92]. A small-molecule modulator called IMU-856 targets sirtuin 6, a transcriptional regulator of intestinal barrier function and regeneration of the bowel epithelium [109]. It was demonstrated in a placebo-controlled Phase Ib study that 28 days of treatment with IMU-856 at two different dose levels (80 and 160 mg), followed by a 15-day gluten challenge with 6 g of gluten daily, significantly attenuated gluten-induced mucosal deterioration as assessed by villous height changes [110].

Research has recently highlighted the functional capacity of the microbiome, particularly at sites of inflammation. In patients with CD, shorter gluten peptides are produced and partially degraded by opportunistic bacteria, such as *Pseudomonas*, which can cross the intestinal barrier more easily and activate T cells. There are several *Lactobacillus* species that are capable of digesting and inactivating both gluten and nongluten amylase/trypsin inhibitor proteins [111,112,113]. So far, clinical studies on probiotics’ effectiveness in CD have been mixed.

In summary, CD is heterogeneous, and it may be difficult to find a treatment that effectively addresses all manifestations. Personalized medicine may be necessary in the future to tailor treatment according to the individual’s needs. These trials offer hope that more convenient, effective, and reliable treatment options will become available for patients with CD in the future. Currently, the GFD remains the only effective treatment option [88].

### 4.3. Screening and Early Detection

The diagnosis of CD can be complicated, as symptoms can vary significantly from patient to patient. As previously mentioned, according to the clinical manifestation, CD is classified into classical, non-classical, subclinical, and refractory [1]. The gold standard for the diagnosis of CD is represented by the combination of mucosal changes and serological test positivity [7,114].

Serology is crucial in diagnosing CD, as it helps to identify individuals who require an intestinal biopsy [73]. It is important to conduct all serological diagnostic tests in patients who are consuming a diet that includes gluten. Anti-tTG-IgA in serum is commonly used for diagnosing CD due to its high sensitivity, although at low levels its specificity decreases [63]. The anti-endomysial IgA antibodies (EMA-IgA) are almost 100% specific for CD, but they are less sensitive, more expensive, and more operator-dependent than the anti-tTG-IgA antibodies. Due to these characteristics, EMA-IgA is an ideal second-line test [63]. Both anti-tTG-IgA and EMA-IgA have a limited diagnostic yield in patients with concurrent IgA deficiency. Moreover, it is important to be cautious when interpreting the low sensitivity of anti-tTG IgG and anti-EMA [37]. Presently, consensus points towards using anti-tTG-IgA as the first serological test, along with checking total IgA levels to eliminate the possibility of IgA deficiency happening at the same time [63]. Finding positive anti-tTG IgA should lead to duodenal biopsies to confirm the diagnosis [115].

Currently, CD cannot be diagnosed using only serology, clinical features, or histology; a combination of them is necessary. In this sense, it is recommended that a sequential approach begins with serology in patients at high risk, followed by a biopsy if the result of serology is positive (together confirming the diagnosis) or if symptoms persist [63]. Although a duodenal biopsy is required in most cases to confirm the diagnosis, it has been suggested to avoid this step in selected children [6]. It is recommended to collect four biopsies from the second duodenal portion and two biopsies from the bulb [116]. Children’s non-biopsy diagnoses have increased so rapidly that many studies consider the increase in serum anti-tTG-IgA (>10× after gluten exposure) as a threshold to further reduce the need for biopsies [117]. CD also has a strong connection with the histocompatibility antigens, such as HLA DQ2 and DQ8. Almost every individual diagnosed with CD will test positive for one or both HLAs or a heterodimer fraction. If there is a discrepancy between serology and histology and in first degree relatives, genetic testing should be performed [73].

It is crucial to emphasize that the diagnosis of potential, silent, and seronegative CD can differ. Potential CD is identified by a positive CD serology test without any mucosal damage on biopsy [118]. In this instance, a diagnosis of CD is confirmed by a serological response around 12 months later [119]. The diagnosis of silent CD is based on a positive serology and histology for CD and the absence of classical or non-classical symptoms [1]. Seronegative CD is identified by active enteropathy, absence of positive serology for CD, ruling out other causes, and showing histological and clinical improvement when following a GFD [1,120]. Before starting a GFD, other causes of enteropathy should be ruled out in these situations. HLA typing can also exclude the possibility of CD in cases of seronegative enteropathies. In order to confirm the diagnosis, a documented histological response after 1–3 years of a GFD is required [119,121,122].

Finally, the diagnosis of refractory CD. To determine the likelihood of refractory CD, the initial diagnosis of CD should be reassessed by examining biopsies and serological tests conducted during the diagnosis process. Individuals with a confirmed diagnosis of CD who adhere strictly to a GFD for over one year and continue to experience unexplained, persistent, or recurring symptoms and/or signs of malabsorption along with damage to the villi in the duodenum are classified as “refractory”. Before determining refractory CD, other related pathological conditions need to be ruled out. The most common of these are intolerances to lactose and fructose, bacterial overgrowth in the small intestine, microscopic colitis, pancreatic insufficiency, and inflammatory bowel disease [63].

## 5. Gluten and Diet

### 5.1. Dietary Management of Celiac Disease

The only effective treatment for CD is the lifelong strict adherence to a GFD. This regimen involves not only the elimination of gluten-containing foods but also vigilance against gluten contamination. However, complete avoidance of gluten re-exposure remains challenging [123]. The difficulty of fully excluding gluten from the diet is reflected in adherence rates, which vary between 42% and 91% across different populations. Suboptimal adherence is influenced by a range of demographic, psychosocial, and clinical factors [124], imposing significant social and financial constraints on patients. Additional challenges to maintaining a GFD include the restrictive nature of the diet, poor palatability, inadequate education and misinformation, inconsistencies in food labeling, and potential cross-contamination [125]. This cross-contamination remains a major issue, occurring both during production and in the preparation of GFFs at home or in restaurants. To support CD patients, it is imperative to provide comprehensive education regarding the adherence to a proper GFD, including guidance on interpreting food labels [126].

Historically, CD management has focused predominantly on avoiding gluten-containing foods, with less emphasis placed on the nutritional quality of the GFD. For optimal health, a GFD must not only be free of gluten but also balanced to meet all energy and nutrient requirements. This includes consuming naturally GFF groups such as fresh fruits, vegetables, seafood, meats, poultry, legumes, nuts, and most dairy products [127]. It is recommended that the diet include daily consumption of 2–3 portions of vegetables, 2–3 portions of fruits, 3–6 portions of gluten-free grains, 2 portions of dairy, and 1–2 portions of protein sources. Additionally, 5–7 portions of nuts and at least 1–2 servings of legumes should be consumed weekly [83].

Research indicates that GFDs are often unbalanced, with a low intake of cereals, fruits, and vegetables, and excessive consumption of meat and derivatives [84]. This imbalance may lead to deficiencies in micro- and macronutrients. The typical GFD often lacks complex carbohydrates and protein while being high in fat and simple carbohydrates [54]. Furthermore, GFFs often lack essential minerals (calcium, copper, iron, magnesium, zinc), vitamins (B12, folate, D), and fiber [128]. Thus, the ideal GFD should be nutrient-dense, balanced in macro- and micronutrients, affordable, and easily accessible [127].

Dietitians play a crucial role in providing effective education on maintaining a strict GFD, and regular follow-up is essential to assess adherence and improve the quality of life for CD patients. Enhancing the access to dietitians, improving tools to assess GFD adherence, increasing the availability of GFFs, and reducing gluten contamination in GFPs are all necessary steps to support CD management [129].

### 5.2. Food Choices and Alternatives

Eliminating all the gluten from the diet poses significant challenges due to the pervasive presence of gluten in the food supply, including the processing of foods that are naturally gluten-free. Moreover, the limitations of testing gluten content and the reliance on manufacturers’ assessments of processed foods further complicate this task [46]. Processed GFPs are often of inferior quality and nutritional value compared to their gluten-containing counterparts [130]. There is a pressing need for greater collaboration among food technologists, nutritionists, the food industry, and regulatory bodies to enhance the nutritional quality of GFPs, thereby improving the health of individuals with CD adhering to a GFD. This includes the exploration of alternative ingredients and technologies to increase the micronutrient and dietary fiber content in GFPs [131].

Furthermore, conventional GFDs tend to have a higher caloric content, often leading to weight gain in patients adhering to a GFD, despite no increase in food intake [54]. A systematic review and meta-analysis of the dietary quality of GFDs revealed that GFFs generally have a lower protein content, which is concerning given protein’s wide-ranging health benefits, and they also tend to have higher fat and salt content compared to their gluten-containing equivalents [131]. The refined flours or starches used in GFPs are typically of poor quality unless fortified with fiber and other nutrients [132].

A broad range of cereals, grains, seeds, legumes, and nuts—such as amaranth, quinoa, millet, sorghum, flax, and chickpeas—can serve as replacements for gluten, offering improved palatability and nutritional quality for GFDs. However, these alternatives are often underutilized, partly due to their high cost and limited availability [133]. The inclusion of oats in a GFD may be beneficial, given their nutritional and health advantages [134]. Gluten-free processed foods made from amaranth, quinoa, and buckwheat offer higher levels of protein, fat, fiber, and minerals compared to those made from rice and corn, making them excellent alternatives to GFPs [135].

**Figure 1:**
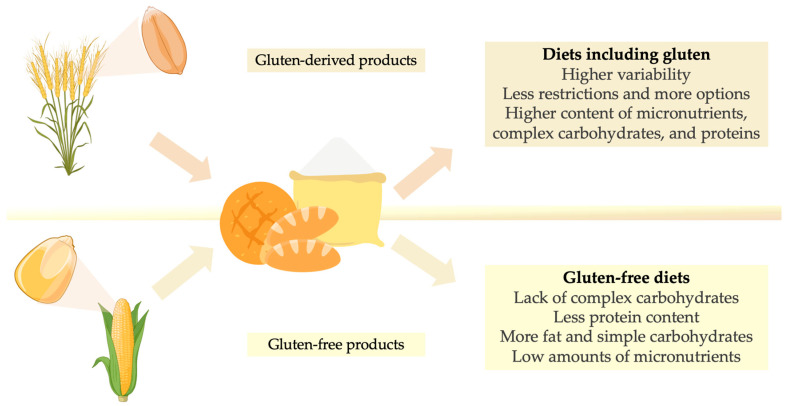
Comparison of diets’ characteristics including cereal products: diets with gluten-derived products vs. gluten-free diets.

Gluten-free options are now available for traditional gluten-containing foods, such as baked goods, using gluten-free cereals and pseudocereals as their base ingredients, such as rice, corn, quinoa, millet, and amaranth [136]. CD patients should consume gluten-free whole grains, pseudocereals, and fortified or enriched products made from these to ensure adequate supplies of complex carbohydrates, protein, fiber, fatty acids, vitamins, and minerals [137]. Pseudocereals, in particular, are predominantly composed of albumins and globulins, containing little to no storage of prolamin proteins, making them excellent substitutes as GFFs [138]. Additionally, amaranth and quinoa are valuable sources of folic acid, riboflavin, vitamin C, and vitamin E [50]. These differences between products with and without gluten have been graphically summarized in Figure 1.

### 5.3. Supplementation in Celiac Disease

In the management of CD, supplementation primarily involves the use of micronutrients, prebiotics, and probiotics. Micronutrient supplementation is critical due to the common deficiencies observed in untreated CD, including calcium, iron, zinc, magnesium, folate, vitamin B12, and vitamin D. These deficiencies can persist even after the adoption of a GFD and should be carefully monitored and addressed [139]. For patients with subclinical or asymptomatic CD, it is recommended to include adequate calcium and vitamin D supplements as part of their GFD regimen, although the evidence supporting this practice is not robust enough [140].

Notably, the effects of supplementation are more pronounced in younger individuals compared to older adults [141]. Supplementation with calcium and vitamin D is particularly important during the first two years of a GFD, as these nutrients play a crucial role in mitigating bone resorption [142]. Iron deficiency, often observed in children with newly diagnosed CD, usually normalizes within 12 months of adhering to a GFD without requiring additional iron supplementation [143]. However, despite strict adherence to a GFD, approximately 30% of CD patients continue to experience nutritional deficiencies, necessitating targeted supplementation [144]. In cases where oral supplementation is ineffective, intravenous administration may be necessary [55]. Zinc levels typically improve with a GFD, independent of zinc supplementation, and deficiencies generally resolve without the need for long-term supplementation [119]. Copper deficiency, though rare, has also been reported in CD and typically normalizes with adequate supplementation and adherence to a GFD [122]. Additionally, B-vitamin supplementation has been shown to effectively reduce homocysteine levels in CD patients, suggesting its potential role in disease management [145].

**Figure 2:**
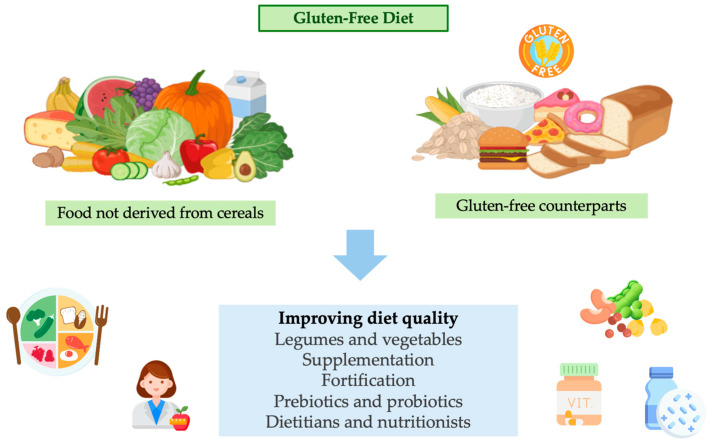
Basis of gluten-free diet and strategies for managing and improving diet quality.

Regarding probiotics and prebiotics, the *Lactobacillus* and *Bifidobacterium* species are the primary probiotics associated with CD management. These microorganisms are present in the intestinal environment and have been found to produce active peptidases capable of breaking down long-chain amino acids, thereby protecting the intestine from severe immunological reactions to gluten [146]. Supplementation with a multispecies probiotic, alongside a GFD, has been deemed safe and may facilitate weight recovery in individuals with growth retardation due to CD [147]. Probiotic supplements are particularly valuable in maintaining a balanced gut microbiome, which may become disrupted following gluten exclusion from the diet [148]. While long-term adherence to a GFD can alleviate many symptoms of CD, intestinal microbiome dysbiosis may persist, and probiotics combined with GFDs have shown promise in restoring the intestinal flora in CD patients, although further research is needed [149]. Prebiotics, such as oligofructose-enriched inulin, are well tolerated in children and adolescents with CD and can enhance iron absorption by decreasing hepcidin levels [150]. Additionally, prebiotics have been shown to improve vitamin D and E status in pediatric CD patients and may offer a novel approach to managing fat-soluble vitamin deficiencies [151]. Despite these positive findings, current clinical guidelines do not universally recommend the use of probiotics, prebiotics, or micronutrient supplementation in the absence of specific nutritional deficiencies or particular clinical conditions [152]. Figure 2 shows the main strategies for the management of CD.

## 6. Socioeconomical Aspects of Celiac Disease

### 6.1. Celiac Disease and Quality of Life

#### 6.1.1. Quality of Life Across Age Groups

In the early stages of life, early diagnosis of CD in asymptomatic patients leads to better quality of life and better compliance with a GFD [153,154,155]. Several studies have shown that children who are lost to follow-up are less likely to adhere to a GFD and have positive celiac antibodies [156]. This underscores the fact that the importance of regular control has been demonstrated.

Nutrition plays an important role in a child’s optimal growth, development, and health [157]. While adhering to a GFD is important for the success of treatment and the prevention of other complications, recent studies indicate that patients who follow this diet may be at risk for nutritional deficiencies. As previously mentioned, GFPs tend to be deficient in fiber, iron, vitamin D, calcium, and have a high glycemic index, contributing to this vulnerability [158,159,160,161]. According to studies, 84% of children and adolescents consume GFPs (which are high in carbohydrates and lipids) between two and three times per day [161,162].

During adulthood, a delay in diagnosis may have a significant impact on the quality of life of patients and may even result in long-term health consequences, such as diminished bone health and malignancy [163,164,165]. It is therefore imperative to identify, diagnose, and treat CD as soon as possible. Currently, the only treatment recommended is lifetime adherence to a GFD, which can reduce symptom severity but can have adverse effects on quality of life [166]. Finally, there may be additional challenges for elderly patients with other coexisting medical conditions and ensuring that a GFD contains a variety of nutrients [83].

#### 6.1.2. Economic Burden

In order to treat CD, patients are required to follow a GFD for the rest of their lives [167]. It is often the case that GFPs are more expensive and less readily available, which increases financial stress and negatively impacts overall well-being [148]. The ideal GFD involves complete elimination of all gluten-containing foods from the diet, including gluten proteins found in barley (hordeins), wheat (gliadins), oats (avenins), rye (secalins), and other closely related grains [168]. One of the main complaints of individuals with CD is the high price of GFPs which it has been broadly confirmed by several studies. This “extra-cost” of GFPs, compared with the gluten-containing counterparts, apparently varies considerably among countries and also among different stores, with reported data ranging from 20% to almost 500%. Furthermore, the increased cost of other food groups not containing gluten (e.g., fruits and vegetables) have an additional indirect cost [169]. Another important aspect is that, although during the past 10 years there has been an important increase in the production and availability of GFPs, this is not sufficient to offset the overall socioeconomic burden for individuals with CD [170].

### 6.2. Psychological and Social Aspects of Living with Celiac Disease

A person with CD may suffer from mood disorders, including manic-depressive disorder, schizophrenia, or bipolar disorder, as well as anxiety and depression [171]. Studies have shown that patients with CD experience low quality of life, anxiety, and depression. Furthermore, they have highlighted the importance of nutrition in reducing these effects [172,173,174,175]. Despite this, very little research has been conducted regarding motivation’s role and impact on quality of life and adherence. As a result of the study’s findings, motivation appears to play a critical role in determining people’s adherence to diet; in fact, motivation was found to be correlated with adherence to diet. Thus, when treating patients with CD, motivation must be taken into account. In addition, it is important to recognize that the constant vigilance required to avoid gluten can lead to frustration, feelings of isolation, and a diminished enjoyment of meals [176].

Taking part in social activities such as dining out, traveling, and attending social events is often challenging due to the limited gluten-free options and the fear of accidental exposure to gluten [177]. According to a study of Dutch patients with CD [178], 23.2% reported food insecurity, and 10.4% reported extremely low food security. A low level of insecurity was associated with poorer quality of diet [178]. There was an association between food insecurity and heightened perceived barriers across several themes, including skills, social circumstances, resources, and GFPs [178]. An analysis of the qualitative data revealed a deeper understanding of these challenges. This included strategies employed to manage costs and insights into the psychological burden associated with GFDs [178].

These disparities are evident across various patient contexts; for instance, children with CD may face challenges during mealtimes at school or social interactions [179], whereas adults with CD may experience difficulties during business lunches or networking events [177].

Comprehensive education on managing a GFD is of great importance, including recognizing hidden gluten sources and navigating social situations [180]. A number of support systems are available, including dietitians, support groups, and mental health professionals, which can ease some of the emotional and social burdens associated with the disease [181]. The availability of GFPs and increased labeling accuracy can simplify daily life for gluten-free patients [182].

### 6.3. Benefits of Physical Exercise in Celiac Disease

Patients with CD experience significant consequences such as malabsorption, which can result in deficits in bone and muscle health due to inadequate absorption of calcium, vitamin D, and other essential nutrients. As a result, patients with untreated or poorly managed CD are at risk of osteoporosis and low bone mineral density [140,183]. Additionally, reduced muscle mass, often observed in patients with CD [184], further exacerbates these risks, as muscle plays a key role in maintaining bone strength and promoting healthy bone mineral density [185] through mechanical loading and endocrine interactions. Regarding fat mass, evidence reveals discrepancies. Some adult patients adhering strictly to a GFD exhibited lower fat mass compared to healthy control groups [184]. However, children with CD had significantly higher mean values of fat-free mass [186]. Consequently, body composition in CD patients differs from that of non-celiac control individuals even after a longstanding GFD [187]. This variation may be partly attributed to unbalanced dietary patterns commonly observed in GFDs, which tend to be higher in fat and lower in carbohydrates. Additionally, a lack of a healthy lifestyle, including regular physical exercise, may further contribute to these differences, underscoring the importance of tailored dietary and lifestyle guidance to support and maintain optimal body composition [188].

Muscle, as one of the most critical endocrine organs, plays a central role in regulating systemic processes, including gut health and immune function [189,190]. Given this, physical exercise emerges as a valuable therapeutic tool for addressing the biopsychosocial impacts of CD. Physical exercise can improve the intestinal mucosa, enhance nutrient absorption, and reduce systemic inflammation, alleviating gastrointestinal symptoms and supporting immune function [189,190,191,192]. Furthermore, physical exercise is known to decrease stress and anxiety while promoting systemic well-being [189,190]. When performed in group settings, it can also foster socialization, addressing some of the psychosocial challenges faced by patients with CD. In addition, the physiological stress and metabolic demands of sports in athletes can negatively impact the quality of life of professional or semi-professional athletes, resulting in a higher risk of developing CD and more severe symptoms than the general population [193].

From a musculoskeletal perspective, 12 weeks of aerobic and resistance training [192], or aerobic training through high-intensity interval training [191], can improve muscle mass and bone health, directly counteracting the detrimental effects of CD on these systems [191,192,194]. Such modes are also linked to better body composition, greater strength, and enhanced gastrointestinal motility, potentially due to favorable changes in the gut microbiome [192,194]. Additionally, physical exercise could increase energy levels, combat fatigue, and improve overall quality of life in patients with CD [189,190]. In children, programs promoting physical activity, particularly increasing time spent in vigorous physical activity, were significantly associated with higher muscle mass and bone mineral density [195].

Although further research is needed to personalize physical exercise recommendations for individuals with CD, its role as a complementary therapy to a GFD is undeniable. Tailored physical exercise programs hold promise in improving not only physical health but also psychological and social well-being in this population.

## 7. Advances, Emerging Therapies, and Future Perspectives

Despite the global advancement of research into this disease, it is vital to facilitate the exchange of knowledge and the undertaking of joint studies, particularly in developing or underdeveloped countries, given the lack of information regarding prevalence and the paucity of clinical studies. Therefore, the establishment of robust international collaboration, enhanced longitudinal care, and the implementation of early screening procedures for children within the general population may offer a potential solution for the development of an effective secondary prevention strategy, with the objective of reducing the social- and health-related burdens. Furthermore, it could facilitate a more comprehensive comprehension of the global prevalence of CD and facilitate the assessment of relevant factors, such as environmental interaction, genotype, or microbiome [9,11].

Currently, there is a great deal of research being conducted in the field of new treatment modalities for CD, and the treatments which are being investigated show potential for meeting the unmet needs of CD patients [88]. Nevertheless, at present, the only effective treatment for CD is the GFD, although increased understanding of the pathophysiology of CD has unveiled different therapeutic targets (see Section 4.2).

CD treatments in development vary not just in their targets but also in their dependence on gluten. Gluten-dependent therapies include glutenases and TG2 inhibitors. These approaches likely require substantial ongoing gluten exposure to demonstrate clinical efficacy, as evidenced by the latiglutenase Phase IIb study (NCT01917630). Gluten-independent approaches, such as IMU-856, or drugs that modulate the immune response, may be effective also in seronegative patients as an adjunct to GFDs or in patients with refractory CD. The observed regenerative properties of IMU-856 in the context of CD suggest that further research might explore its potential application in other gastrointestinal disorders as well [196]. In accordance with the FDA’s draft guidance for CD, gluten-independent treatments may be particularly relevant, as the guidance emphasizes drug development as an adjunct to GFDs for patients exhibiting signs and symptoms of the disease despite strict dietary adherence, rather than drugs that substitute for GFDs. It is important to note that certain types of treatments are tailored for specific subtypes of CD, such as refractory CD, where treatments such as IL-15 inhibitors target the aberrant immune response characteristic of the more severe form of CD that is not associated with gluten consumption.

## 8. Conclusions

Despite the growing knowledge of CD and the awareness of patients suffering from this condition, the management of dietary aspects remains a challenge. Among the main remarkable problems are the high prevalence of undiagnosed cases, a higher cost and lower availability of gluten free options, the risk of cross-contamination, products with lower nutritional quality in comparison with their gluten-containing counterparts with the consequent risk of malnutrition, changes in gut microbiota composition linked to GFDs, and the lack of public policies to support CD patients. Research is needed to better understand and manage this disease, as well as a greater commitment by the food industry, governments, and institutions in order to improve the quality of life of these patients.

## References

[1] Ludvigsson J.F., Leffler D.A., Bai J.C., Biagi F., Fasano A., Green P.H., Hadjivassiliou M., Kaukinen K., Kelly C.P., Leonard J.N. (2013). The Oslo definitions for coeliac disease and related terms. Gut.

[2] Lebwohl B., Rubio-Tapia A. (2021). Epidemiology, Presentation, and Diagnosis of Celiac Disease. Gastroenterology.

[3] Ye L., Zheng W., Li X., Han W., Shen J., Lin Q., Hou L., Liao L., Zeng X. (2023). The Role of Gluten in Food Products and Dietary Restriction: Exploring the Potential for Restoring Immune Tolerance. Foods.

[4] Shewry P. (2019). What Is Gluten-Why Is It Special?. Front. Nutr..

[5] Schuster C., Huen J., Weiss T., Scherf K.A. (2024). Rapid analysis of wheat gluten composition using a triple ELISA. J. Sci. Food Agric..

[6] Rubio-Tapia A., Hill I.D., Semrad C., Kelly C.P., Greer K.B., Limketkai B.N., Lebwohl B. (2023). American College of Gastroenterology Guidelines Update: Diagnosis and Management of Celiac Disease. Am. J. Gastroenterol..

[7] Caio G., Volta U., Sapone A., Leffler D.A., De Giorgio R., Catassi C., Fasano A. (2019). Celiac disease: A comprehensive current review. BMC Med..

[8] Auricchio R., Calabrese I., Galatola M., Cielo D., Carbone F., Mancuso M., Matarese G., Troncone R., Auricchio S., Greco L. (2022). Gluten consumption and inflammation affect the development of celiac disease in at-risk children. Sci. Rep..

[9] Demir E., Comba A. (2020). The evolution of celiac disease publications: A holistic approach with bibliometric analysis. Ir. J. Med. Sci..

[10] Serena G., D’Avino P., Fasano A. (2020). Celiac Disease and Non-celiac Wheat Sensitivity: State of Art of Non-dietary Therapies. Front. Nutr..

[11] Gatti S., Rubio-Tapia A., Makharia G., Catassi C. (2024). Patient and Community Health Global Burden in a World with More Celiac Disease. Gastroenterology.

[12] Mohta S., Rajput M.S., Ahuja V., Makharia G.K. (2021). Emergence of Celiac disease and Gluten-related disorders in Asia. J. Neurogastroenterol. Motil..

[13] Singh P., Arora A., Strand T.A., Leffler D.A., Catassi C., Green P.H., Kelly C.P., Ahuja V., Makharia G.K. (2018). Global Prevalence of Celiac Disease: Systematic Review and Meta-analysis. Clin. Gastroenterol. Hepatol..

[14] King J.A., Jeong J., Underwood F.E., Quan J., Panaccione N., Windsor J.W., Coward S., deBruyn J., Ronksley P.E., Shaheen A.A. (2020). Incidence of Celiac Disease Is Increasing Over Time: A Systematic Review and Meta-analysis. Am. J. Gastroenterol..

[15] Choung R.S., Ditah I.C., Nadeau A.M., Rubio-Tapia A., Marietta E.V., Brantner T.L., Camilleri M.J., Rajkumar S.V., Landgren O., Everhart J.E. (2015). Trends and racial/ethnic disparities in gluten-sensitive problems in the United States: Findings from the National Health and Nutrition Examination Surveys from 1988 to 2012. Am. J. Gastroenterol..

[16] Zingone F., Bai J.C., Cellier C., Ludvigsson J.F. (2024). Celiac Disease-Related Conditions: Who to Test?. Gastroenterology.

[17] Aggarwal N., Agarwal A., Alarouri H., Dwarakanathan V., Dang S., Ahuja V., Makharia G.K. (2024). Patients with Celiac Disease Have High Prevalence of Fatty Liver and Metabolic Syndrome. Dig. Dis. Sci..

[18] Li T., Feng Y., Wang C., Shi T., Huang X., Abuduhadeer M., Abudurexiti A., Zhang M., Gao F. (2024). Causal relationships between autoimmune diseases and celiac disease: A Mendelian randomization analysis. Biotechnol. Genet. Eng. Rev..

[19] Shuler B., Liu E., Stahl M.G. (2023). Population level screening for celiac disease: Is now the time?. Curr. Opin. Gastroenterol..

[20] Lebwohl B., Sanders D.S., Green P.H.R. (2018). Coeliac disease. Lancet.

[21] France A., Penmetsa A. (2024). A Review of Celiac Disease and Its Implications on Fertility and Pregnancy. Semin. Reprod. Med..

[22] Sahin Y. (2021). Celiac disease in children: A review of the literature. World J. Clin. Pediatr..

[23] Ching C.K., Lebwohl B. (2022). Celiac Disease in the Elderly. Curr. Treat. Options Gastroenterol..

[24] Bishop J., Ravikumara M. (2020). Coeliac disease in childhood: An overview. J. Paediatr. Child. Health.

[25] Szajewska H., Shamir R., Chmielewska A., Piescik-Lech M., Auricchio R., Ivarsson A., Kolacek S., Koletzko S., Korponay-Szabo I., Mearin M.L. (2015). Systematic review with meta-analysis: Early infant feeding and coeliac disease—Update 2015. Aliment. Pharmacol. Ther..

[26] Lionetti E., Castellaneta S., Francavilla R., Pulvirenti A., Tonutti E., Amarri S., Barbato M., Barbera C., Barera G., Bellantoni A. (2014). Introduction of gluten, HLA status, and the risk of celiac disease in children. N. Engl. J. Med..

[27] Ludvigsson J.F., Card T.R., Kaukinen K., Bai J., Zingone F., Sanders D.S., Murray J.A. (2015). Screening for celiac disease in the general population and in high-risk groups. United Eur. Gastroenterol. J..

[28] Arvanitakis K., Siargkas A., Germanidis G., Dagklis T., Tsakiridis I. (2023). Adverse pregnancy outcomes in women with celiac disease: A systematic review and meta-analysis. Ann. Gastroenterol..

[29] Pinto-Sanchez M.I., Silvester J.A., Lebwohl B., Leffler D.A., Anderson R.P., Therrien A., Kelly C.P., Verdu E.F. (2021). Society for the Study of Celiac Disease position statement on gaps and opportunities in coeliac disease. Nat. Rev. Gastroenterol. Hepatol..

[30] Sapone A., Bai J.C., Ciacci C., Dolinsek J., Green P.H., Hadjivassiliou M., Kaukinen K., Rostami K., Sanders D.S., Schumann M. (2012). Spectrum of gluten-related disorders: Consensus on new nomenclature and classification. BMC Med..

[31] Taraghikhah N., Ashtari S., Asri N., Shahbazkhani B., Al-Dulaimi D., Rostami-Nejad M., Rezaei-Tavirani M., Razzaghi M.R., Zali M.R. (2020). An updated overview of spectrum of gluten-related disorders: Clinical and diagnostic aspects. BMC Gastroenterol..

[32] Pasha I., Saeed F., Sultan M.T., Batool R., Aziz M., Ahmed W. (2016). Wheat Allergy and Intolerence; Recent Updates and Perspectives. Crit. Rev. Food Sci. Nutr..

[33] Iversen R., Sollid L.M. (2023). The Immunobiology and Pathogenesis of Celiac Disease. Annu. Rev. Pathol..

[34] Sollid L.M. (2002). Coeliac disease: Dissecting a complex inflammatory disorder. Nat. Rev. Immunol..

[35] De Re V., Magris R., Cannizzaro R. (2017). New Insights into the Pathogenesis of Celiac Disease. Front. Med..

[36] McAllister B.P., Williams E., Clarke K. (2019). A Comprehensive Review of Celiac Disease/Gluten-Sensitive Enteropathies. Clin. Rev. Allergy Immunol..

[37] Calado J., Verdelho Machado M. (2022). Celiac Disease Revisited. GE Port. J. Gastroenterol..

[38] Marsilio I., Maddalo G., Ghisa M., Savarino E.V., Farinati F., Zingone F. (2020). The coeliac stomach: A review of the literature. Dig. Liver Dis..

[39] Wieser H., Ciacci C., Soldaini C., Gizzi C., Santonicola A. (2024). Gastrointestinal and Hepatobiliary Manifestations Associated with Untreated Celiac Disease in Adults and Children: A Narrative Overview. J. Clin. Med..

[40] Montoro-Huguet M.A., Belloc B., Dominguez-Cajal M. (2021). Small and Large Intestine (I): Malabsorption of Nutrients. Nutrients.

[41] Panezai M.S., Ullah A., Ballur K., Gilstrap L., Khan J., Tareen B., Kakar M., Khan J., Rasheed A., Waheed A. (2021). Frequency of Celiac Disease in Patients with Chronic Diarrhea. Cureus.

[42] Wieser H., Ciacci C., Gizzi C., Santonicola A. (2023). Otorhinolaryngological Manifestations and Esophageal Disorders in Celiac Disease: A Narrative Review. J. Clin. Med..

[43] Kunovsky L., Dite P., Jabandziev P., Eid M., Poredska K., Vaculova J., Sochorova D., Janecek P., Tesarikova P., Blaho M. (2021). Causes of Exocrine Pancreatic Insufficiency Other Than Chronic Pancreatitis. J. Clin. Med..

[44] Hoffmanova I., Sanchez D., Tuckova L., Tlaskalova-Hogenova H. (2018). Celiac Disease and Liver Disorders: From Putative Pathogenesis to Clinical Implications. Nutrients.

[45] Narciso-Schiavon J.L., Schiavon L.L. (2017). To screen or not to screen? Celiac antibodies in liver diseases. World J. Gastroenterol..

[46] Pinto-Sanchez M.I., Blom J.J., Gibson P.R., Armstrong D. (2024). Nutrition Assessment and Management in Celiac Disease. Gastroenterology.

[47] Gholmie Y., Lee A.R., Satherley R.M., Schebendach J., Zybert P., Green P.H.R., Lebwohl B., Wolf R. (2023). Maladaptive Food Attitudes and Behaviors in Individuals with Celiac Disease and Their Association with Quality of Life. Dig. Dis. Sci..

[48] Lavrisa Z., Hribar M., Kusar A., Zmitek K., Pravst I. (2020). Nutritional Composition of Gluten-Free Labelled Foods in the Slovenian Food Supply. Int. J. Environ. Res. Public Health.

[49] Seron-Arbeloa C., Labarta-Monzon L., Puzo-Foncillas J., Mallor-Bonet T., Lafita-Lopez A., Bueno-Vidales N., Montoro-Huguet M. (2022). Malnutrition Screening and Assessment. Nutrients.

[50] Di Nardo G., Villa M.P., Conti L., Ranucci G., Pacchiarotti C., Principessa L., Raucci U., Parisi P. (2019). Nutritional Deficiencies in Children with Celiac Disease Resulting from a Gluten-Free Diet: A Systematic Review. Nutrients.

[51] Allen B., Orfila C. (2018). The Availability and Nutritional Adequacy of Gluten-Free Bread and Pasta. Nutrients.

[52] Jivraj A., Hutchinson J.M., Ching E., Marwaha A., Verdu E.F., Armstrong D., Pinto-Sanchez M.I. (2022). Micronutrient deficiencies are frequent in adult patients with and without celiac disease on a gluten-free diet, regardless of duration and adherence to the diet. Nutrition.

[53] Elli L., Leffler D., Cellier C., Lebwohl B., Ciacci C., Schumann M., Lundin K.E.A., Chetcuti Zammit S., Sidhu R., Roncoroni L. (2024). Guidelines for best practices in monitoring established coeliac disease in adult patients. Nat. Rev. Gastroenterol. Hepatol..

[54] Abdi F., Zuberi S., Blom J.J., Armstrong D., Pinto-Sanchez M.I. (2023). Nutritional Considerations in Celiac Disease and Non-Celiac Gluten/Wheat Sensitivity. Nutrients.

[55] Catassi C., Verdu E.F., Bai J.C., Lionetti E. (2022). Coeliac disease. Lancet.

[56] Rossi R.E., Masoni B., Zullo A., De Deo D., Hassan C., Repici A. (2024). Clinical presentation of celiac disease in adult patients: Current real-life experience. Intern. Emerg. Med..

[57] Laurikka P., Nurminen S., Kivela L., Kurppa K. (2018). Extraintestinal Manifestations of Celiac Disease: Early Detection for Better Long-Term Outcomes. Nutrients.

[58] Santonicola A., Wieser H., Gizzi C., Soldaini C., Ciacci C. (2024). Associations between Celiac Disease, Extra-Gastrointestinal Manifestations, and Gluten-Free Diet: A Narrative Overview. Nutrients.

[59] Therrien A., Kelly C.P., Silvester J.A. (2020). Celiac Disease: Extraintestinal Manifestations and Associated Conditions. J. Clin. Gastroenterol..

[60] Hujoel I.A., Murray J.A. (2020). Refractory Celiac Disease. Curr. Gastroenterol. Rep..

[61] Eigner W., Bashir K., Primas C., Kazemi-Shirazi L., Wrba F., Trauner M., Vogelsang H. (2017). Dynamics of occurrence of refractory coeliac disease and associated complications over 25 years. Aliment. Pharmacol. Ther..

[62] Celli R., Hui P., Triscott H., Bogardus S., Gibson J., Hwang M., Robert M.E. (2019). Clinical Insignficance of Monoclonal T-Cell Populations and Duodenal Intraepithelial T-Cell Phenotypes in Celiac and Nonceliac Patients. Am. J. Surg. Pathol..

[63] Raiteri A., Granito A., Giamperoli A., Catenaro T., Negrini G., Tovoli F. (2022). Current guidelines for the management of celiac disease: A systematic review with comparative analysis. World J. Gastroenterol..

[64] Wieser H., Amato M., Caggiano M., Ciacci C. (2023). Dental Manifestations and Celiac Disease—An Overview. J. Clin. Med..

[65] Fousekis F.S., Katsanos A., Katsanos K.H., Christodoulou D.K. (2020). Ocular manifestations in celiac disease: An overview. Int. Ophthalmol..

[66] Vats V., Makineni P., Hemaida S., Haider A., Subramani S., Kaur N., Butt A.N., Scott-Emuakpor R., Zahir M., Mathew M. (2023). Gluten Intolerance and Its Association with Skin Disorders: A Narrative Review. Cureus.

[67] Kondapalli A.V., Walker M.D. (2022). Celiac disease and bone. Arch. Endocrinol. Metab..

[68] Evangelatos G., Kouna K., Iliopoulos A., Fragoulis G.E. (2023). Musculoskeletal Complications of Celiac Disease: A Case-Based Review. Mediterr. J. Rheumatol..

[69] Habura I., Fiedorowicz K., Wozniak A., Idasiak-Piechocka I., Kosikowski P., Oko A. (2019). IgA nephropathy associated with coeliac disease. Cent. Eur. J. Immunol..

[70] Pelizzaro F., Marsilio I., Fassan M., Piazza F., Barberio B., D’Odorico A., Savarino E.V., Farinati F., Zingone F. (2021). The Risk of Malignancies in Celiac Disease—A Literature Review. Cancers.

[71] Ivanova M., Bottiglieri L., Sajjadi E., Venetis K., Fusco N. (2023). Malignancies in Patients with Celiac Disease: Diagnostic Challenges and Molecular Advances. Genes.

[72] Vanoli A., Di Sabatino A., Furlan D., Klersy C., Grillo F., Fiocca R., Mescoli C., Rugge M., Nesi G., Fociani P. (2017). Small Bowel Carcinomas in Coeliac or Crohn’s Disease: Clinico-pathological, Molecular, and Prognostic Features. A Study From the Small Bowel Cancer Italian Consortium. J. Crohns Colitis.

[73] Villanacci V., Vanoli A., Leoncini G., Arpa G., Salviato T., Bonetti L.R., Baronchelli C., Saragoni L., Parente P. (2020). Celiac disease: Histology-differential diagnosis-complications. A practical approach. Pathologica.

[74] Kamboj A.K., Oxentenko A.S. (2017). Clinical and Histologic Mimickers of Celiac Disease. Clin. Transl. Gastroenterol..

[75] Schiepatti A., Cincotta M., Biagi F., Sanders D.S. (2021). Enteropathies with villous atrophy but negative coeliac serology in adults: Current issues. BMJ Open Gastroenterol..

[76] Ensari A. (2010). Gluten-sensitive enteropathy (celiac disease): Controversies in diagnosis and classification. Arch. Pathol. Lab. Med..

[77] Hanevik K., Wik E., Langeland N., Hausken T. (2018). Transient elevation of anti-transglutaminase and anti-endomysium antibodies in Giardia infection. Scand. J. Gastroenterol..

[78] Rubio-Tapia A., Herman M.L., Ludvigsson J.F., Kelly D.G., Mangan T.F., Wu T.T., Murray J.A. (2012). Severe spruelike enteropathy associated with olmesartan. Mayo Clin. Proc..

[79] Esposito G., Dottori L., Pivetta G., Ligato I., Dilaghi E., Lahner E. (2022). Pernicious Anemia: The Hematological Presentation of a Multifaceted Disorder Caused by Cobalamin Deficiency. Nutrients.

[80] Fernandes L., Machado B., Jose Cruz A., Sarmento G., Quelhas Costa R., Pereira T., Scigliano H., Cerqueira R. (2023). Collagenous sprue: A rare cause of watery diarrhea and villous atrophy—Case report. Gastroenterol. Hepatol. Bed Bench.

[81] Anderson R.P., Verma R., Schumann M. (2024). A Look Into the Future: Are We Ready for an Approved Therapy in Celiac Disease?. Gastroenterology.

[82] Maimaris S., Schiepatti A., Biagi F. (2024). Systematic review with meta-analysis: Cause-specific and all-cause mortality trends across different coeliac disease phenotypes. Aliment. Pharmacol. Ther..

[83] Simon E., Molero-Luis M., Fueyo-Diaz R., Costas-Batlle C., Crespo-Escobar P., Montoro-Huguet M.A. (2023). The Gluten-Free Diet for Celiac Disease: Critical Insights to Better Understand Clinical Outcomes. Nutrients.

[84] Cardo A., Churruca I., Lasa A., Navarro V., Vazquez-Polo M., Perez-Junkera G., Larretxi I. (2021). Nutritional Imbalances in Adult Celiac Patients Following a Gluten-Free Diet. Nutrients.

[85] Estevez V., Rodriguez J.M., Schlack P., Navarrete P., Bascunan K.A., Nunez V., Oyarce C., Flores C., Ayala J., Araya M. (2024). Persistent Barriers of the Gluten-Free Basic Food Basket: Availability, Cost, and Nutritional Composition Assessment. Nutrients.

[86] Ribeiro C.D.S., Uenishi R.H., Domingues A.D.S., Nakano E.Y., Botelho R.B.A., Raposo A., Zandonadi R.P. (2024). Gluten-Free Diet Adherence Tools for Individuals with Celiac Disease: A Systematic Review and Meta-Analysis of Tools Compared to Laboratory Tests. Nutrients.

[87] Harnett J.E., Myers S.P. (2020). Quality of life in people with ongoing symptoms of coeliac disease despite adherence to a strict gluten-free diet. Sci. Rep..

[88] Dieckman T., Koning F., Bouma G. (2022). Celiac disease: New therapies on the horizon. Curr. Opin. Pharmacol..

[89] Murray J.A., Syage J.A., Wu T.T., Dickason M.A., Ramos A.G., Van Dyke C., Horwath I., Lavin P.T., Maki M., Hujoel I. (2022). Latiglutenase Protects the Mucosa and Attenuates Symptom Severity in Patients with Celiac Disease Exposed to a Gluten Challenge. Gastroenterology.

[90] Pultz I.S., Hill M., Vitanza J.M., Wolf C., Saaby L., Liu T., Winkle P., Leffler D.A. (2021). Gluten Degradation, Pharmacokinetics, Safety, and Tolerability of TAK-062, an Engineered Enzyme to Treat Celiac Disease. Gastroenterology.

[91] Sample D.A., Sunwoo H.H., Huynh H.Q., Rylance H.L., Robert C.L., Xu B.W., Kang S.H., Gujral N., Dieleman L.A. (2017). AGY, a Novel Egg Yolk-Derived Anti-gliadin Antibody, Is Safe for Patients with Celiac Disease. Dig. Dis. Sci..

[92] Burianek F., Gege C., Marinkovic P. (2024). New developments in celiac disease treatments. Drug Discov. Today.

[93] Konig J., Holster S., Bruins M.J., Brummer R.J. (2017). Randomized clinical trial: Effective gluten degradation by Aspergillus niger-derived enzyme in a complex meal setting. Sci. Rep..

[94] Salden B.N., Monserrat V., Troost F.J., Bruins M.J., Edens L., Bartholome R., Haenen G.R., Winkens B., Koning F., Masclee A.A. (2015). Randomised clinical study: Aspergillus niger-derived enzyme digests gluten in the stomach of healthy volunteers. Aliment. Pharmacol. Ther..

[95] Murray J.A., Kelly C.P., Green P.H.R., Marcantonio A., Wu T.T., Maki M., Adelman D.C., CeliAction Study Group of Investigators (2017). No Difference Between Latiglutenase and Placebo in Reducing Villous Atrophy or Improving Symptoms in Patients with Symptomatic Celiac Disease. Gastroenterology.

[96] Montserrat V., Bruins M.J., Edens L., Koning F. (2015). Influence of dietary components on Aspergillus niger prolyl endoprotease mediated gluten degradation. Food Chem..

[97] Wei G., Helmerhorst E.J., Darwish G., Blumenkranz G., Schuppan D. (2020). Gluten Degrading Enzymes for Treatment of Celiac Disease. Nutrients.

[98] Paolella G., Sposito S., Romanelli A.M., Caputo I. (2022). Type 2 Transglutaminase in Coeliac Disease: A Key Player in Pathogenesis, Diagnosis and Therapy. Int. J. Mol. Sci..

[99] Levescot A., Malamut G., Cerf-Bensussan N. (2022). Immunopathogenesis and environmental triggers in coeliac disease. Gut.

[100] Schuppan D., Maki M., Lundin K.E.A., Isola J., Friesing-Sosnik T., Taavela J., Popp A., Koskenpato J., Langhorst J., Hovde O. (2021). A Randomized Trial of a Transglutaminase 2 Inhibitor for Celiac Disease. N. Engl. J. Med..

[101] Sollid L.M. (2024). Tolerance-inducing therapies in coeliac disease—Mechanisms, progress and future directions. Nat. Rev. Gastroenterol. Hepatol..

[102] Camarca A., Rotondi Aufiero V., Mazzarella G. (2023). Role of Regulatory T Cells and Their Potential Therapeutic Applications in Celiac Disease. Int. J. Mol. Sci..

[103] Truitt K.E., Daveson A.J.M., Ee H.C., Goel G., MacDougall J., Neff K., Anderson R.P. (2019). Randomised clinical trial: A placebo-controlled study of subcutaneous or intradermal NEXVAX2, an investigational immunomodulatory peptide therapy for coeliac disease. Aliment. Pharmacol. Ther..

[104] Daveson A.J.M., Ee H.C., Andrews J.M., King T., Goldstein K.E., Dzuris J.L., MacDougall J.A., Williams L.J., Treohan A., Cooreman M.P. (2017). Epitope-Specific Immunotherapy Targeting CD4-Positive T Cells in Celiac Disease: Safety, Pharmacokinetics, and Effects on Intestinal Histology and Plasma Cytokines with Escalating Dose Regimens of Nexvax2 in a Randomized, Double-Blind, Placebo-Controlled Phase 1 Study. eBioMedicine.

[105] Goel G., King T., Daveson A.J., Andrews J.M., Krishnarajah J., Krause R., Brown G.J.E., Fogel R., Barish C.F., Epstein R. (2017). Epitope-specific immunotherapy targeting CD4-positive T cells in coeliac disease: Two randomised, double-blind, placebo-controlled phase 1 studies. Lancet Gastroenterol. Hepatol..

[106] Kelly C.P., Murray J.A., Leffler D.A., Getts D.R., Bledsoe A.C., Smithson G., First M.R., Morris A., Boyne M., Elhofy A. (2021). TAK-101 Nanoparticles Induce Gluten-Specific Tolerance in Celiac Disease: A Randomized, Double-Blind, Placebo-Controlled Study. Gastroenterology.

[107] Abadie V., Kim S.M., Lejeune T., Palanski B.A., Ernest J.D., Tastet O., Voisine J., Discepolo V., Marietta E.V., Hawash M.B.F. (2020). IL-15, gluten and HLA-DQ8 drive tissue destruction in coeliac disease. Nature.

[108] Lahdeaho M.L., Scheinin M., Vuotikka P., Taavela J., Popp A., Laukkarinen J., Koffert J., Koivurova O.P., Pesu M., Kivela L. (2019). Safety and efficacy of AMG 714 in adults with coeliac disease exposed to gluten challenge: A phase 2a, randomised, double-blind, placebo-controlled study. Lancet Gastroenterol. Hepatol..

[109] Liu G., Chen H., Liu H., Zhang W., Zhou J. (2021). Emerging roles of SIRT6 in human diseases and its modulators. Med. Res. Rev..

[110] Daveson A.J.M., Stubbs R., Polasek T.M., Isola J., Anderson R., Tye-Din J.A., Schoeman M., Lionnet C., Mei S., Mihajlovic J. (2025). Safety, clinical activity, pharmacodynamics, and pharmacokinetics of IMU-856, a SIRT6 modulator, in coeliac disease: A first-in-human, randomised, double-blind, placebo-controlled, phase 1 trial. Lancet Gastroenterol. Hepatol..

[111] Galipeau H.J., Verdu E.F. (2022). The double-edged sword of gut bacteria in celiac disease and implications for therapeutic potential. Mucosal Immunol..

[112] Verdu E.F., Schuppan D. (2021). Co-factors, Microbes, and Immunogenetics in Celiac Disease to Guide Novel Approaches for Diagnosis and Treatment. Gastroenterology.

[113] Sanz Y. (2010). Effects of a gluten-free diet on gut microbiota and immune function in healthy adult humans. Gut Microbes.

[114] Villanacci V., Del Sordo R., Casella G., Becheanu G., Oberti A., Belfekih B., Bassotti G., Ravelli A. (2023). The correct methodological approach to the diagnosis of celiac disease: The point of view of the pathologist. Gastroenterol. Hepatol. Bed Bench.

[115] Kowalski K., Mulak A., Jasinska M., Paradowski L. (2017). Diagnostic challenges in celiac disease. Adv. Clin. Exp. Med..

[116] Pais W.P., Duerksen D.R., Pettigrew N.M., Bernstein C.N. (2008). How many duodenal biopsy specimens are required to make a diagnosis of celiac disease?. Gastrointest. Endosc..

[117] Badizadegan K., Vanlandingham D.M., Hampton W., Thompson K.M. (2020). Value of biopsy in a cohort of children with high-titer celiac serologies: Observation of dynamic policy differences between Europe and North America. BMC Health Serv. Res..

[118] Shiha M.G., Schiepatti A., Maimaris S., Nandi N., Penny H.A., Sanders D.S. (2024). Clinical outcomes of potential coeliac disease: A systematic review and meta-analysis. Gut.

[119] Al-Toma A., Volta U., Auricchio R., Castillejo G., Sanders D.S., Cellier C., Mulder C.J., Lundin K.E.A. (2019). European Society for the Study of Coeliac Disease (ESsCD) guideline for coeliac disease and other gluten-related disorders. United Eur. Gastroenterol. J..

[120] Poddighe D., Abdukhakimova D. (2021). Celiac Disease in Asia beyond the Middle East and Indian subcontinent: Epidemiological burden and diagnostic barriers. World J. Gastroenterol..

[121] Leonard M.M., Lebwohl B., Rubio-Tapia A., Biagi F. (2021). AGA Clinical Practice Update on the Evaluation and Management of Seronegative Enteropathies: Expert Review. Gastroenterology.

[122] Rubio-Tapia A., Hill I.D., Kelly C.P., Calderwood A.H., Murray J.A., American College of Gastroenterology (2013). ACG clinical guidelines: Diagnosis and management of celiac disease. Am. J. Gastroenterol..

[123] Colella M., Cafiero C., Palmirotta R. (2024). Aspergillus niger prolyl endopeptidase in celiac disease. World J. Gastroenterol..

[124] Mulder C.J.J., Elli L., Lebwohl B., Makharia G.K., Rostami K., Rubio-Tapia A., Schumann M., Tye-Din J., Zeitz J., Al-Toma A. (2023). Follow-Up of Celiac Disease in Adults: “When, What, Who, and Where”. Nutrients.

[125] Selimoglu M.A., Karabiber H. (2010). Celiac disease: Prevention and treatment. J. Clin. Gastroenterol..

[126] Marasco G., Cirota G.G., Rossini B., Lungaro L., Di Biase A.R., Colecchia A., Volta U., De Giorgio R., Festi D., Caio G. (2020). Probiotics, Prebiotics and Other Dietary Supplements for Gut Microbiota Modulation in Celiac Disease Patients. Nutrients.

[127] Theethira T.G., Dennis M. (2015). Celiac disease and the gluten-free diet: Consequences and recommendations for improvement. Dig. Dis..

[128] Diez-Sampedro A., Olenick M., Maltseva T., Flowers M. (2019). A Gluten-Free Diet, Not an Appropriate Choice without a Medical Diagnosis. J. Nutr. Metab..

[129] Makharia G.K., Singh P., Catassi C., Sanders D.S., Leffler D., Ali R.A.R., Bai J.C. (2022). The global burden of coeliac disease: Opportunities and challenges. Nat. Rev. Gastroenterol. Hepatol..

[130] Kreutz J.M., Adriaanse M.P.M., van der Ploeg E.M.C., Vreugdenhil A.C.E. (2020). Narrative Review: Nutrient Deficiencies in Adults and Children with Treated and Untreated Celiac Disease. Nutrients.

[131] Melini V., Melini F. (2019). Gluten-Free Diet: Gaps and Needs for a Healthier Diet. Nutrients.

[132] Rai S., Kaur A., Chopra C.S. (2018). Gluten-Free Products for Celiac Susceptible People. Front. Nutr..

[133] Bascunan K.A., Vespa M.C., Araya M. (2017). Celiac disease: Understanding the gluten-free diet. Eur. J. Nutr..

[134] Comino I., Moreno Mde L., Sousa C. (2015). Role of oats in celiac disease. World J. Gastroenterol..

[135] Alvarez-Jubete L., Arendt E.K., Gallagher E. (2009). Nutritive value and chemical composition of pseudocereals as gluten-free ingredients. Int. J. Food Sci. Nutr..

[136] El Khoury D., Balfour-Ducharme S., Joye I.J. (2018). A Review on the Gluten-Free Diet: Technological and Nutritional Challenges. Nutrients.

[137] Lee A.R., Ng D.L., Dave E., Ciaccio E.J., Green P.H. (2009). The effect of substituting alternative grains in the diet on the nutritional profile of the gluten-free diet. J. Hum. Nutr. Diet..

[138] Hosseini S.M., Soltanizadeh N., Mirmoghtadaee P., Banavand P., Mirmoghtadaie L., Shojaee-Aliabadi S. (2018). Gluten-free products in celiac disease: Nutritional and technological challenges and solutions. J. Res. Med. Sci..

[139] Caruso R., Pallone F., Stasi E., Romeo S., Monteleone G. (2013). Appropriate nutrient supplementation in celiac disease. Ann. Med..

[140] Duerksen D., Pinto-Sanchez M.I., Anca A., Schnetzler J., Case S., Zelin J., Smallwood A., Turner J., Verdu E., Butzner J.D. (2018). Management of bone health in patients with celiac disease: Practical guide for clinicians. Can. Fam. Physician.

[141] Capriles V.D., Martini L.A., Areas J.A. (2009). Metabolic osteopathy in celiac disease: Importance of a gluten-free diet. Nutr. Rev..

[142] Zanchetta M.B., Longobardi V., Bai J.C. (2016). Bone and Celiac Disease. Curr. Osteoporos. Rep..

[143] Ben-Ami T., Trotskovsky A., Topf-Olivestone C., Kori M. (2024). Iron deficiency without anemia in children with newly diagnosed celiac disease: 1-year follow-up of ferritin levels, with and without iron supplementation. Eur. J. Pediatr..

[144] Alkalay M.J. (2021). Nutrition in Patients with Lactose Malabsorption, Celiac Disease, and Related Disorders. Nutrients.

[145] Hadithi M., Mulder C.J., Stam F., Azizi J., Crusius J.B., Pena A.S., Stehouwer C.D., Smulders Y.M. (2009). Effect of B vitamin supplementation on plasma homocysteine levels in celiac disease. World J. Gastroenterol..

[146] Moawad M.H., Alkhawaldeh I.M., Naswhan A.J. (2023). Efficacy of probiotics supplementation in amelioration of celiac disease symptoms and enhancement of immune system. World J. Clin. Cases.

[147] Lionetti E., Dominijanni V., Iasevoli M., Cimadamore E., Acquaviva I., Gatti S., Monachesi C., Catassi G., Pino A., Faragalli A. (2023). Effects of the supplementation with a multispecies probiotic on clinical and laboratory recovery of children with newly diagnosed celiac disease: A randomized, placebo-controlled trial. Dig. Liver Dis..

[148] Aljada B., Zohni A., El-Matary W. (2021). The Gluten-Free Diet for Celiac Disease and Beyond. Nutrients.

[149] Wu X., Qian L., Liu K., Wu J., Shan Z. (2021). Gastrointestinal microbiome and gluten in celiac disease. Ann. Med..

[150] Ferus K., Drabinska N., Krupa-Kozak U., Jarocka-Cyrta E. (2018). A Randomized, Placebo-Controlled, Pilot Clinical Trial to Evaluate the Effect of Supplementation with Prebiotic Synergy 1 on Iron Homeostasis in Children and Adolescents with Celiac Disease Treated with a Gluten-Free Diet. Nutrients.

[151] Drabinska N., Krupa-Kozak U., Abramowicz P., Jarocka-Cyrta E. (2018). Beneficial Effect of Oligofructose-Enriched Inulin on Vitamin D and E Status in Children with Celiac Disease on a Long-Term Gluten-Free Diet: A Preliminary Randomized, Placebo-Controlled Nutritional Intervention Study. Nutrients.

[152] McDermid J.M., Almond M.A., Roberts K.M., Germer E.M., Geller M.G., Taylor T.A., Sinley R.C., Handu D. (2023). Celiac Disease: An Academy of Nutrition and Dietetics Evidence-Based Nutrition Practice Guideline. J. Acad. Nutr. Diet..

[153] Krauthammer A., Guz-Mark A., Zevit N., Marderfeld L., Waisbourd-Zinman O., Silbermintz A., Mozer-Glassberg Y., Nachmias Friedler V., Rozenfeld Bar Lev M., Matar M. (2020). Two decades of pediatric celiac disease in a tertiary referral center: What has changed?. Dig. Liver Dis..

[154] Mahadev S., Gardner R., Lewis S.K., Lebwohl B., Green P.H. (2016). Quality of Life in Screen-detected Celiac Disease Patients in the United States. J. Clin. Gastroenterol..

[155] Webb C., Myleus A., Norstrom F., Hammarroth S., Hogberg L., Lagerqvist C., Rosen A., Sandstrom O., Stenhammar L., Ivarsson A. (2015). High adherence to a gluten-free diet in adolescents with screening-detected celiac disease. J. Pediatr. Gastroenterol. Nutr..

[156] Barnea L., Mozer-Glassberg Y., Hojsak I., Hartman C., Shamir R. (2014). Pediatric celiac disease patients who are lost to follow-up have a poorly controlled disease. Digestion.

[157] Macedo L., Catarino M., Festas C., Alves P. (2024). Vulnerability in Children with Celiac Disease: Findings from a Scoping Review. Children.

[158] Nayar S., Mahapatra S. Nutritional intake, gluten-free diet compliance and quality of life of pediatric patients with celiac disease. Proceedings of the II International Symposium on Medicinal and Nutraceutical Plants 972.

[159] Scaramuzza A.E., Mantegazza C., Bosetti A., Zuccotti G.V. (2013). Type 1 diabetes and celiac disease: The effects of gluten free diet on metabolic control. World J. Diabetes.

[160] Al-Majali M.A., Burayzat S., Tayyem R.F. (2023). Dietary Management of Type 1 Diabetes Mellitus with Celiac Disease. Curr. Diabetes Rev..

[161] Fernández C.B., Varela-Moreiras G., Úbeda N., Alonso-Aperte E. (2019). Nutritional status in Spanish children and adolescents with celiac disease on a gluten free diet compared to non-celiac disease controls. Nutrients.

[162] Alzaben A.S., Turner J., Shirton L., Samuel T.M., Persad R., Mager D. (2015). Assessing nutritional quality and adherence to the gluten-free diet in children and adolescents with celiac disease. Can. J. Diet. Pract. Res..

[163] Laurikka P., Kivelä L., Kurppa K., Kaukinen K. (2022). Systemic consequences of coeliac disease. Aliment. Pharmacol. Ther..

[164] Fuchs V., Kurppa K., Huhtala H., Mäki M., Kekkonen L., Kaukinen K. (2018). Delayed celiac disease diagnosis predisposes to reduced quality of life and incremental use of health care services and medicines: A prospective nationwide study. United Eur. Gastroenterol. J..

[165] Gray A.M., Papanicolas I.N. (2010). Impact of symptoms on quality of life before and after diagnosis of coeliac disease: Results from a UK population survey. BMC Health Serv. Res..

[166] Lee A.R. (2022). Dietary management of coeliac disease. Aliment. Pharmacol. Ther..

[167] Itzlinger A., Branchi F., Elli L., Schumann M. (2018). Gluten-Free Diet in Celiac Disease-Forever and for All?. Nutrients.

[168] Niland B., Cash B.D. (2018). Health benefits and adverse effects of a gluten-free diet in non–celiac disease patients. Gastroenterol. Hepatol..

[169] Bozorg S.R., Lee A.R., Marild K., Murray J.A. (2024). The Economic Iceberg of Celiac Disease: More Than the Cost of Gluten-Free Food. Gastroenterology.

[170] Lee A.R., Wolf R.L., Lebwohl B., Ciaccio E.J., Green P.H.R. (2019). Persistent Economic Burden of the Gluten Free Diet. Nutrients.

[171] Alkhiari R. (2023). Psychiatric and Neurological Manifestations of Celiac Disease in Adults. Cureus.

[172] Guedes N.G., Silva L.A.D., Bessa C.C., Santos J.C.D., Silva V.M.D., Lopes M.V.O. (2020). Anxiety and depression: A study of psychoaffective, family-related, and daily-life factors in celiac individuals. Rev. Bras. Enferm..

[173] Wolf R.L., Lebwohl B., Lee A.R., Zybert P., Reilly N.R., Cadenhead J., Amengual C., Green P.H.R. (2018). Hypervigilance to a Gluten-Free Diet and Decreased Quality of Life in Teenagers and Adults with Celiac Disease. Dig. Dis. Sci..

[174] Zysk W., Glabska D., Guzek D. (2018). Social and Emotional Fears and Worries Influencing the Quality of Life of Female Celiac Disease Patients Following a Gluten-Free Diet. Nutrients.

[175] Zingone F., Swift G.L., Card T.R., Sanders D.S., Ludvigsson J.F., Bai J.C. (2015). Psychological morbidity of celiac disease: A review of the literature. United Eur. Gastroenterol. J..

[176] Barberis N., Quattropani M.C., Cuzzocrea F. (2019). Relationship between motivation, adherence to diet, anxiety symptoms, depression symptoms and quality of life in individuals with celiac disease. J. Psychosom. Res..

[177] Leffler D.A., Edwards-George J., Dennis M., Schuppan D., Cook F., Franko D.L., Blom-Hoffman J., Kelly C.P. (2008). Factors that influence adherence to a gluten-free diet in adults with celiac disease. Dig. Dis. Sci..

[178] Smeets S.M., Kiefte-de Jong J.C., van der Velde L.A. (2024). Food insecurity and other barriers to adherence to a gluten-free diet in individuals with celiac disease and non-celiac gluten sensitivity in the Netherlands: A mixed-methods study. medRxiv.

[179] Meyer S., Rosenblum S. (2017). Activities, Participation and Quality of Life Concepts in Children and Adolescents with Celiac Disease: A Scoping Review. Nutrients.

[180] Mazzola A.M., Zammarchi I., Valerii M.C., Spisni E., Saracino I.M., Lanzarotto F., Ricci C. (2024). Gluten-Free Diet and Other Celiac Disease Therapies: Current Understanding and Emerging Strategies. Nutrients.

[181] Lee A.R., Dennis M., Lebovits J., Welstead L., Verma R., Therrien A., Lebwohl B. (2024). Dietary assessments in individuals living with coeliac disease: Key considerations. J. Hum. Nutr. Diet..

[182] Osorio C.E., Mejias J.H., Rustgi S. (2019). Gluten Detection Methods and Their Critical Role in Assuring Safe Diets for Celiac Patients. Nutrients.

[183] Fedewa M.V., Bentley J.L., Higgins S., Kindler J.M., Esco M.R., MacDonald H.V. (2020). Celiac Disease and Bone Health in Children and Adolescents: A Systematic Review and Meta-Analysis. J. Clin. Densitom..

[184] Bardella M.T., Fredella C., Prampolini L., Molteni N., Giunta A.M., Bianchi P.A. (2000). Body composition and dietary intakes in adult celiac disease patients consuming a strict gluten-free diet. Am. J. Clin. Nutr..

[185] Skoracka K., Michalak M., Ratajczak-Pawłowska A.E., Rychter A.M., Zawada A., Dobrowolska A., Krela-Kaźmierczak I. (2024). The Effect of Body Composition on Osteoporosis Risk in Adults with Celiac Disease. Gastroenterol. Insights.

[186] Wiech P., Chmiel Z., Bazalinski D., Salacinska I., Bartosiewicz A., Mazur A., Korczowski B., Binkowska-Bury M., Dabrowski M. (2018). The Relationship between Body Composition and a Gluten Free Diet in Children with Celiac Disease. Nutrients.

[187] Vereczkei Z., Farkas N., Hegyi P., Imrei M., Foldi M., Szakacs Z., Kiss S., Solymar M., Nagy R., Bajor J. (2021). It Is High Time for Personalized Dietary Counseling in Celiac Disease: A Systematic Review and Meta-Analysis on Body Composition. Nutrients.

[188] Pelc A., Walicka-Cuprys K., Puszkarz G., Stys K., Chmiel E., Wilk S., Ludwikowski G., Placek K. (2024). Evaluation of the relationship between body composition and dietary habits of physically active people with disabilities. Sci. Rep..

[189] Suzuki K. (2019). Chronic Inflammation as an Immunological Abnormality and Effectiveness of Exercise. Biomolecules.

[190] Motiani K.K., Collado M.C., Eskelinen J.J., Virtanen K.A., Löyttyniemi E., Salminen S., Nuutila P., Kalliokoski K.K., Hannukainen J.C. (2020). Exercise Training Modulates Gut Microbiota Profile and Improves Endotoxemia. Med. Sci. Sports Exerc..

[191] Dowd A.J., Kronlund L., Warbeck C., Parmar C., Daun J.T., Wytsma-Fisher K., Reimer R.A., Millet G., Fung T., Culos-Reed S.N. (2022). Effects of a 12-week HIIT + group mediated cognitive behavioural intervention on quality of life among inactive adults with coeliac disease: Findings from the pilot MOVE-C study. Psychol. Health.

[192] Martínez-Rodríguez A., Loaiza-Martínez D.A., Sánchez-Sánchez J., Rubio-Arias J.A., Alacid F., Prats-Moya S., Martínez-Olcina M., Yáñez-Sepúlveda R., Asencio-Mas N., Marcos-Pardo P.J. (2021). Effects of 12 weeks of strength training and gluten-free diet on quality of life, body composition and strength in women with celiac disease: A randomized controlled trial. Appl. Sci..

[193] Leone J.E., Wise K.A., Mullin E.M., Gray K.A., Szlosek P.A., Griffin M.F., Jordan C.A. (2020). Celiac Disease Symptoms in Athletes: Prevalence Indicators of Perceived Quality of Life. Sports Health.

[194] Nestares T., Martín-Masot R., de Teresa C., Bonillo R., Maldonado J., Flor-Alemany M., Aparicio V.A. (2021). Influence of Mediterranean Diet Adherence and Physical Activity on Bone Health in Celiac Children on a Gluten-Free Diet. Nutrients.

[195] Clauss M., Gérard P., Mosca A., Leclerc M. (2021). Interplay between exercise and gut microbiome in the context of human health and performance. Front. Nutr..

[196] Muehler A., Slizgi J.R., Kohlhof H., Groeppel M., Peelen E., Vitt D. (2020). Clinical relevance of intestinal barrier dysfunction in common gastrointestinal diseases. World J. Gastrointest. Pathophysiol..

